# Osteoid Osteoma of the Calcaneus in a Young Patient Treated With Radiofrequency Ablation: A Case Report

**DOI:** 10.7759/cureus.48521

**Published:** 2023-11-08

**Authors:** Sultan K Alharbi, Abdulrahman M Alaseem, Wessal A Alhomaied, Rana Z Alsaeed, Abdulaziz M AlSudairi, Yazeed A Alsehibani

**Affiliations:** 1 Department of Orthopedics, Majmaah University, Riyadh, SAU; 2 Department of Orthopedic Surgery, College of Medicine, King Saud University, Riyadh, SAU; 3 Department of Orthopedic Surgery, King Abdullah bin Abdulaziz University Hospital, Riyadh, SAU; 4 Department of Orthopedic Surgery, College of Medicine, Princess Nourah Bint Abdulrahman University, Riyadh, SAU

**Keywords:** functional and clinical outcome, calcaneus, osteoid osteoma, case report, radiofrequency ablation (rfa)

## Abstract

Osteoid osteoma (OO) is a common benign tumor that tends to affect children and young adults. Patients typically present with nocturnal pain that is relieved with non-steroidal anti-inflammatory drugs (NSAIDs) and a unique round or oval radiolucent area with surrounding sclerotic bone on X-ray. The cortex of the diaphysis or metaphysis of long bones is the usual anatomical location, with only 4% of cases localizing to the foot and ankle. Treatment options include medical management, surgical excision, and less invasive techniques such as radiofrequency ablation (RFA). We present a case report of a 21-year-old female with an osteoid osteoma of the calcaneus, a rare presentation for this type of tumor. She was successfully treated with RFA and had an excellent functional outcome.

## Introduction

Osteoid osteoma (OO) is a common benign tumor that accounts for 11% of benign tumors and 5% of all bone tumors [1]. It tends to affect children and young adults with characteristic clinical and radiographic appearance. Patients typically present with nocturnal pain that is relieved with non-steroidal anti-inflammatory drugs (NSAIDs) [2]. A distinguishing radiological feature is a round or oval radiolucent area with surrounding sclerotic bone [3]. Osteoid osteomas (OOs) usually arise in the cortex of the diaphysis or metaphysis of long bones, with only 4% of cases localizing to the foot and ankle [2]. Besides the rarity of hindfoot presentation, it carries several challenges. They are often misdiagnosed as the presentation mimics other more common diseases and radiographically shows less reactive sclerosis [4,5]. Treatment options include medical management, surgical excision, and less invasive techniques such as radiofrequency ablation (RFA), which yielded good outcomes [1].

The current case report describes a 21-year-old female with an osteoid osteoma of the calcaneus, a rare presentation for this type of tumor. She was successfully treated with RFA and had an excellent functional outcome.

## Case presentation

**Figure 1:**
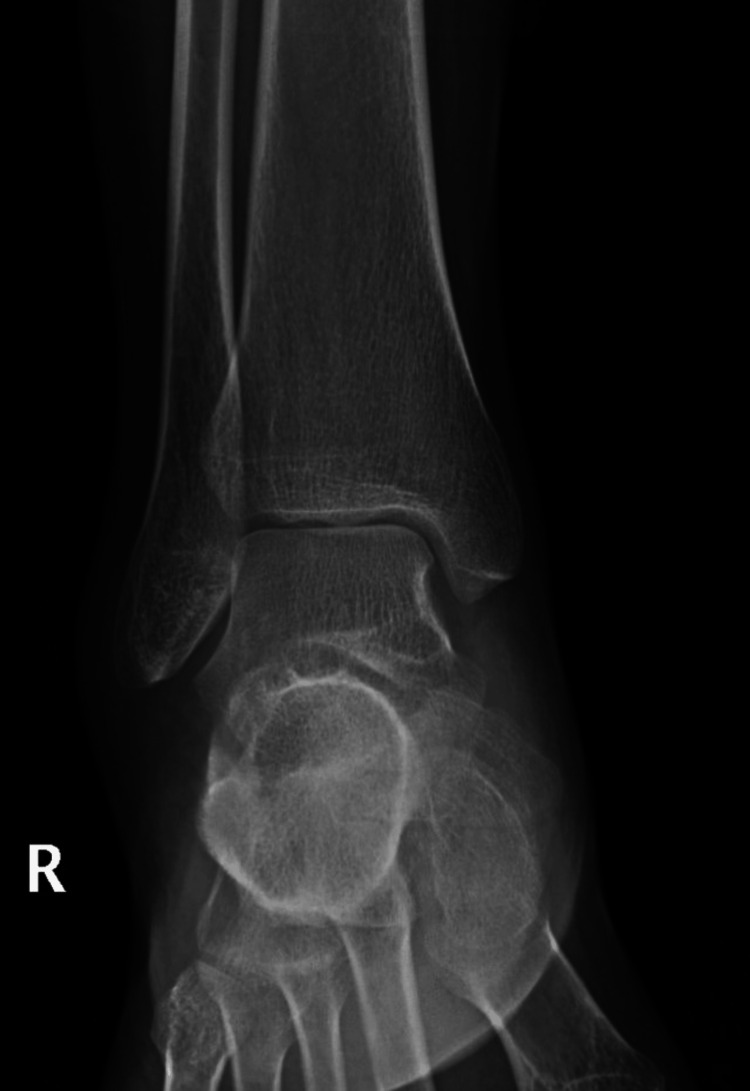
Anteroposterior view X-ray of the right ankle at the time of presentation

**Figure 2:**
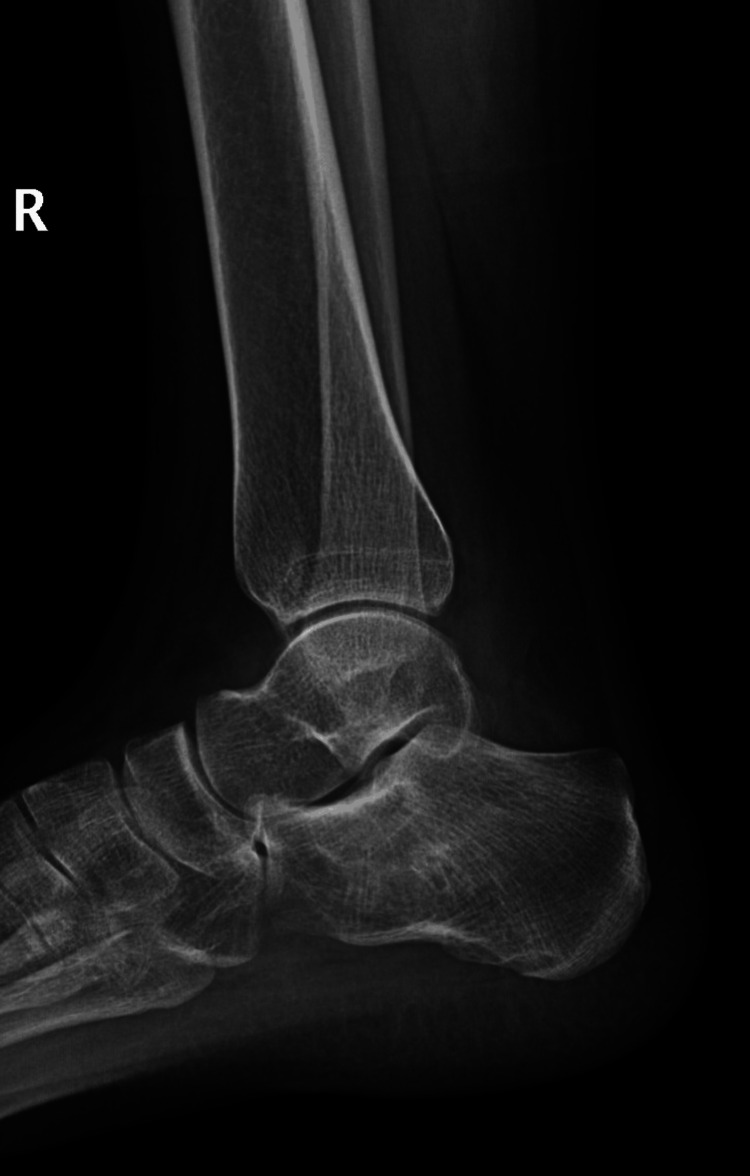
Lateral view X-ray of the right ankle at the time of presentation

**Figure 3:**
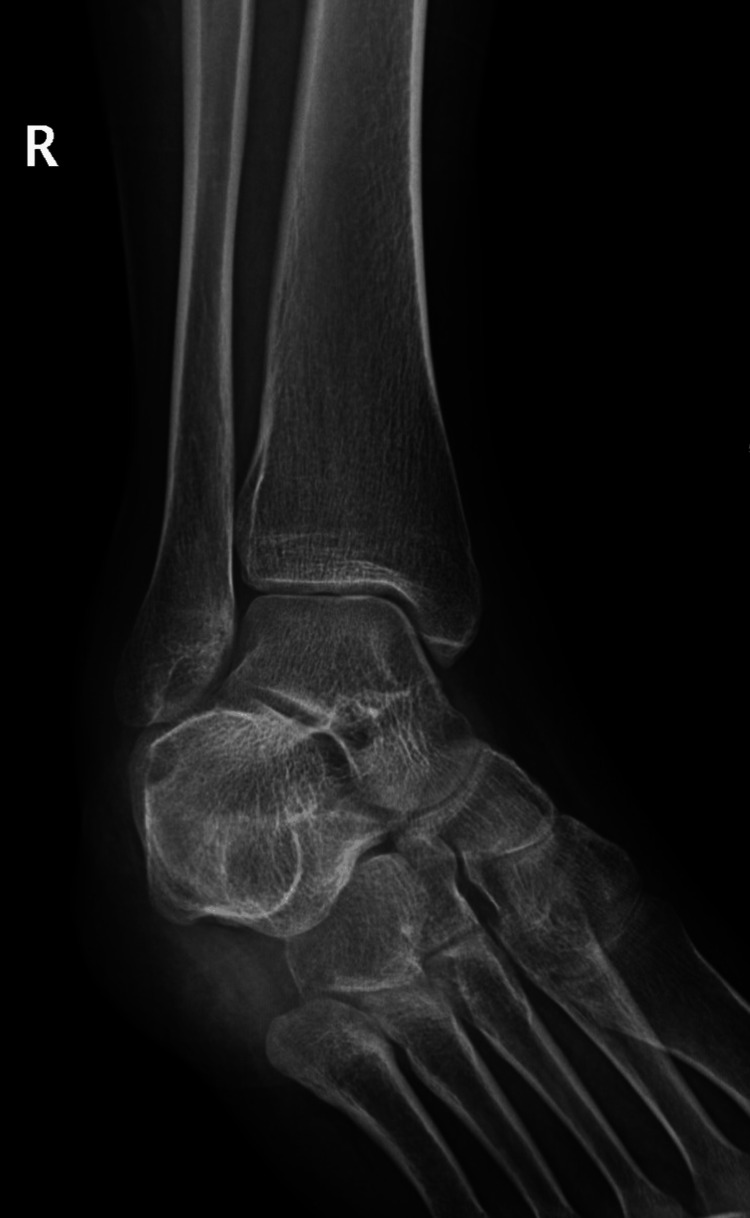
Mortise view X-ray of the right ankle at the time of presentation

**Figure 4:**
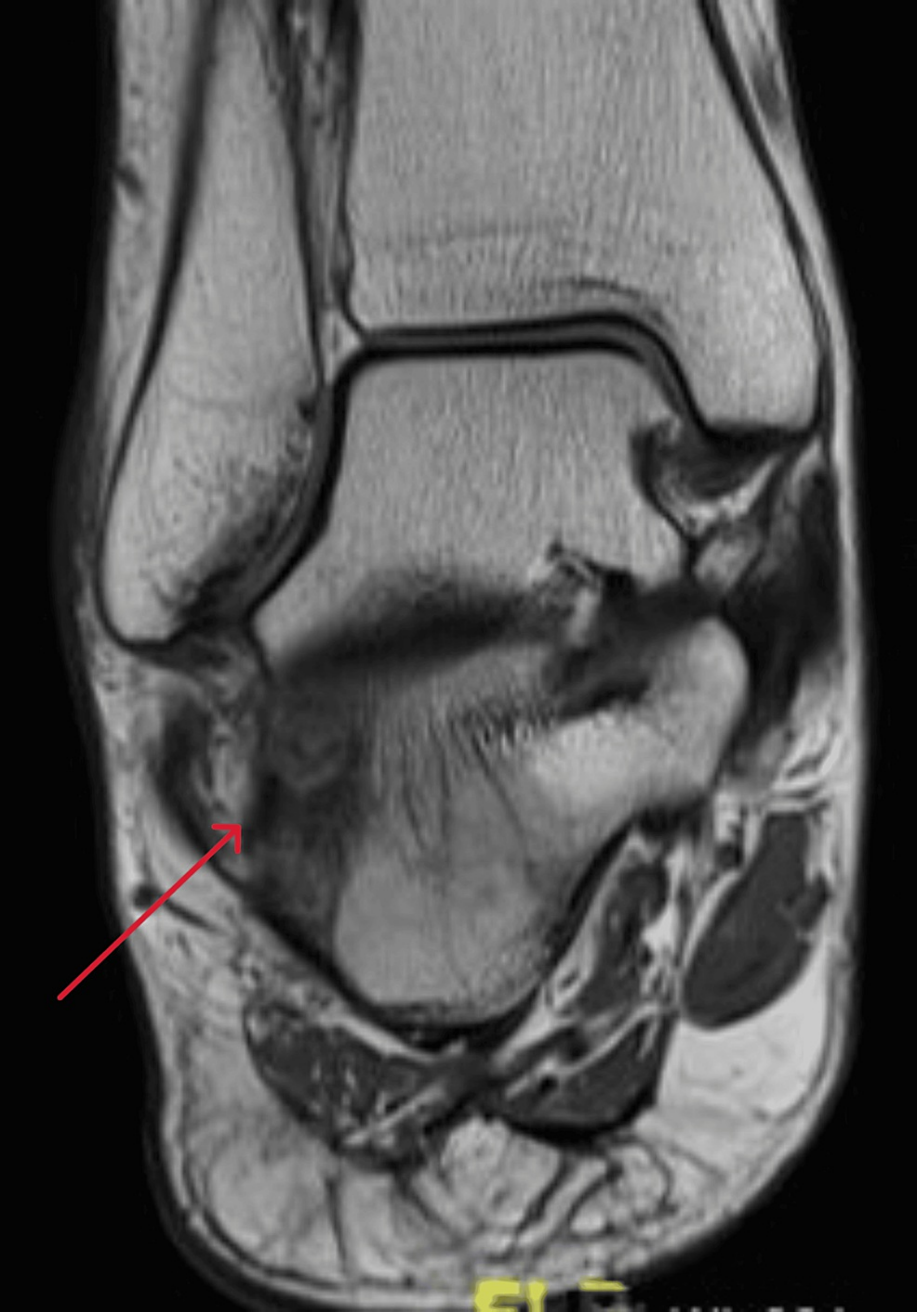
Coronal view MRI T1 window of the right ankle at the time of presentation MRI: magnetic resonance imaging

**Figure 5:**
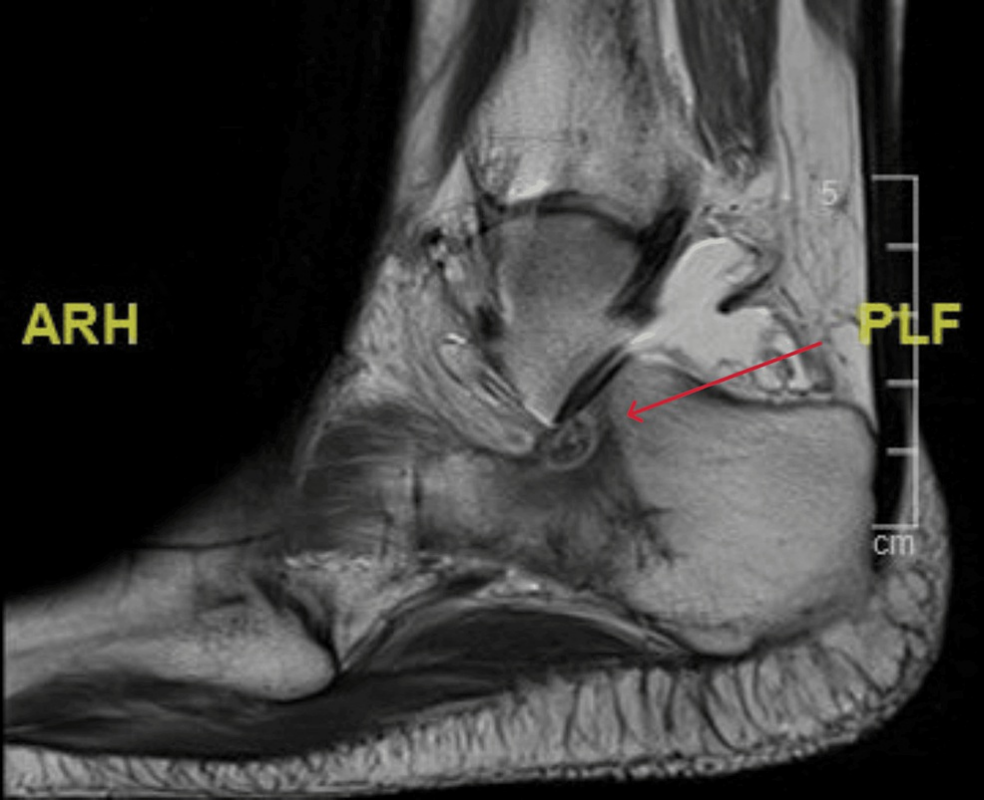
Sagittal view MRI T1 window of the right ankle at the time of presentation MRI: magnetic resonance imaging

**Figure 6:**
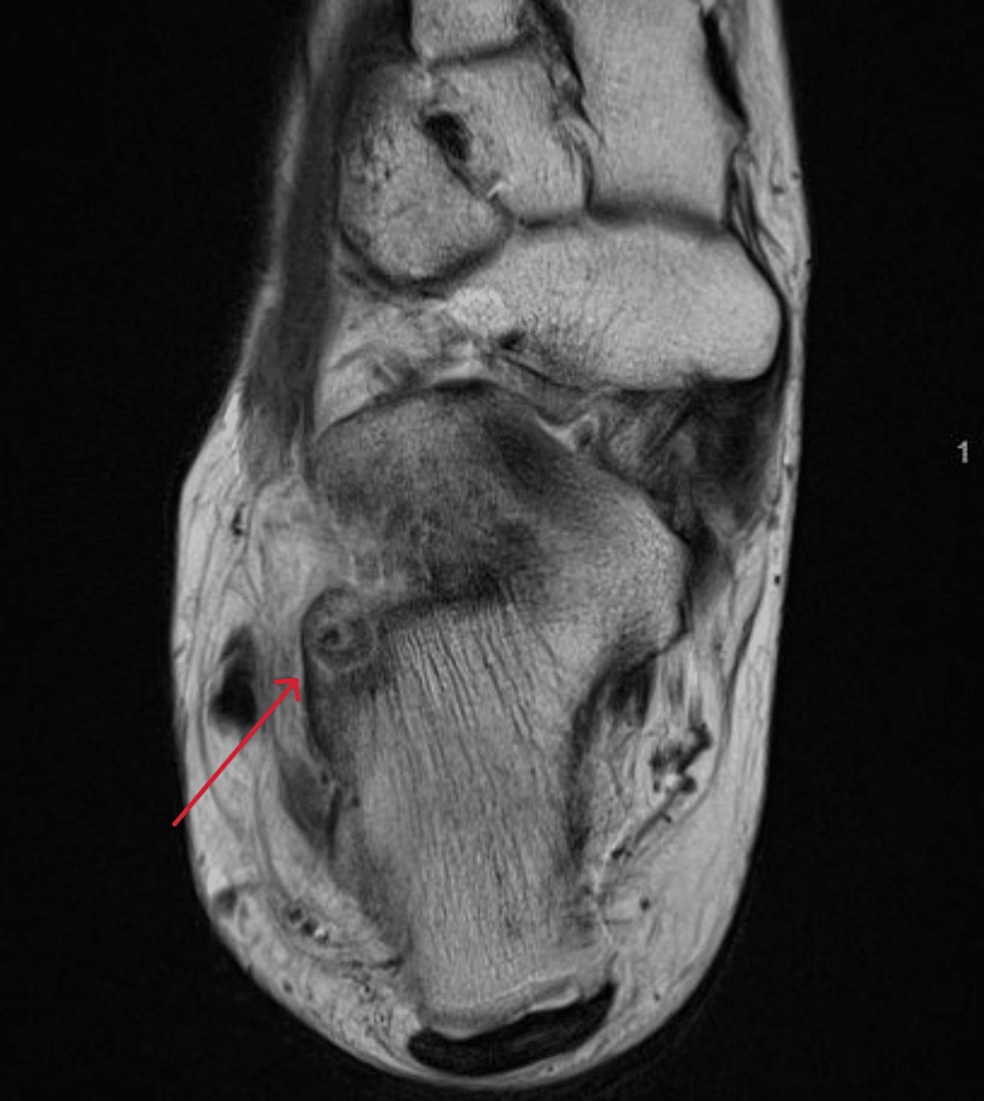
Axial view MRI T1 window of the right ankle at the time of presentation MRI: magnetic resonance imaging

**Figure 7:**
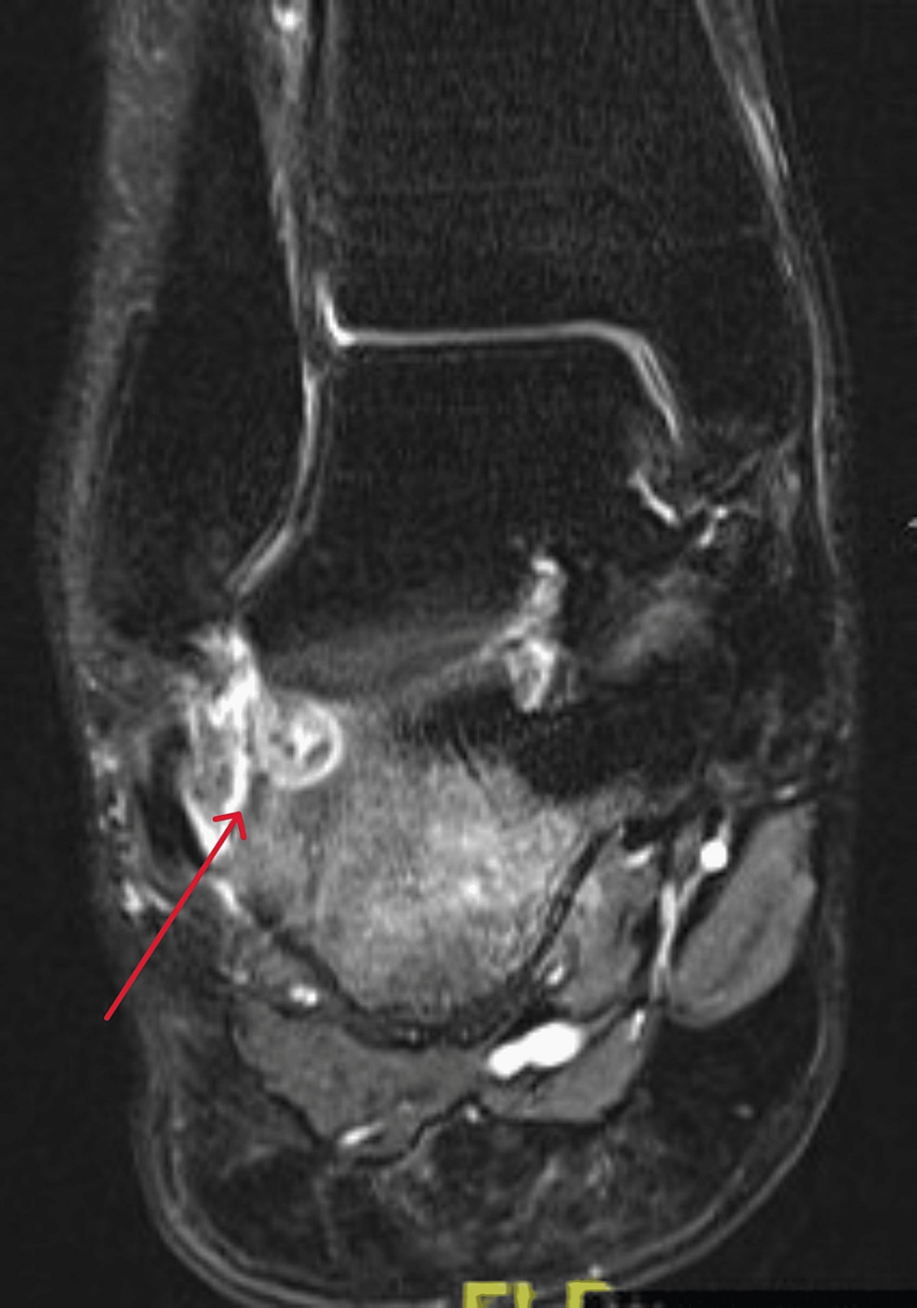
Coronal view MRI T2 window of the right ankle at the time of presentation MRI: magnetic resonance imaging

**Figure 8:**
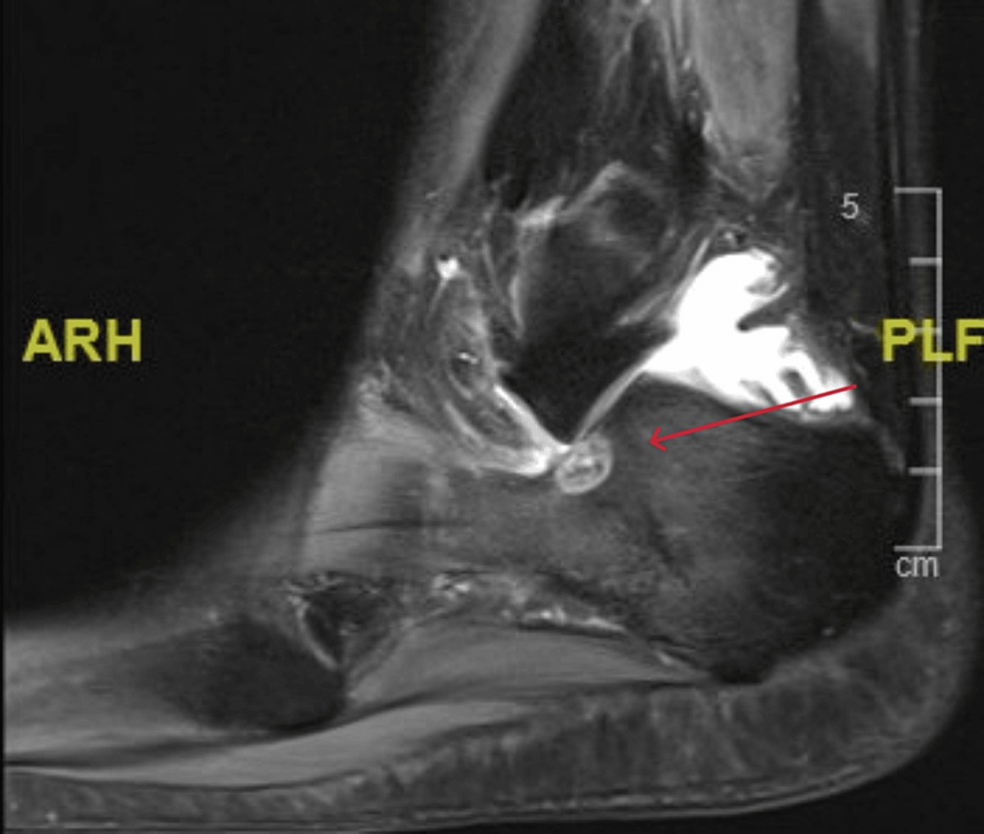
Sagittal view MRI T2 window of the right ankle at the time of presentation MRI: magnetic resonance imaging

**Figure 9:**
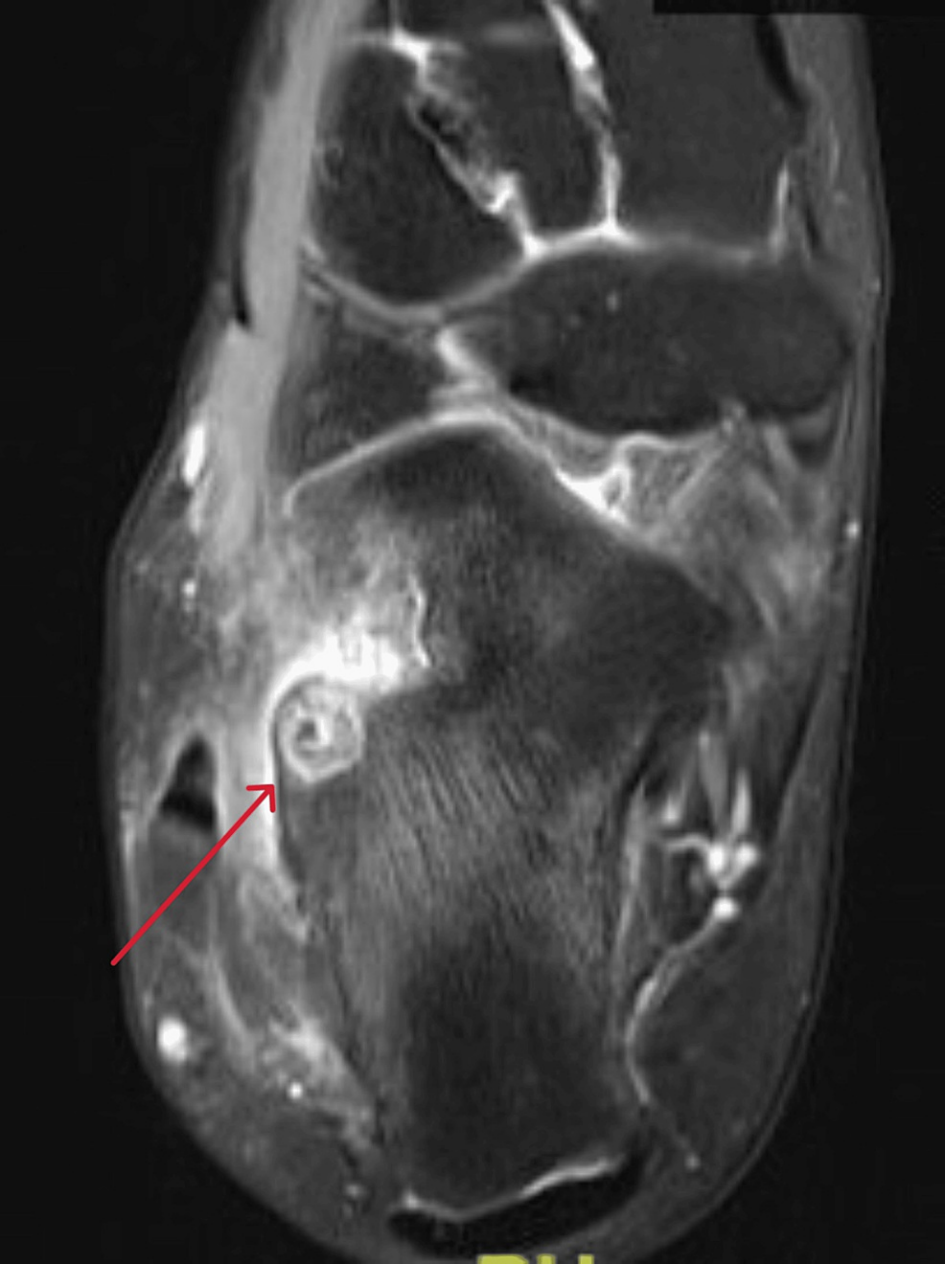
Axial view MRI T2 window of the right ankle at the time of presentation MRI: magnetic resonance imaging

**Figure 10:**
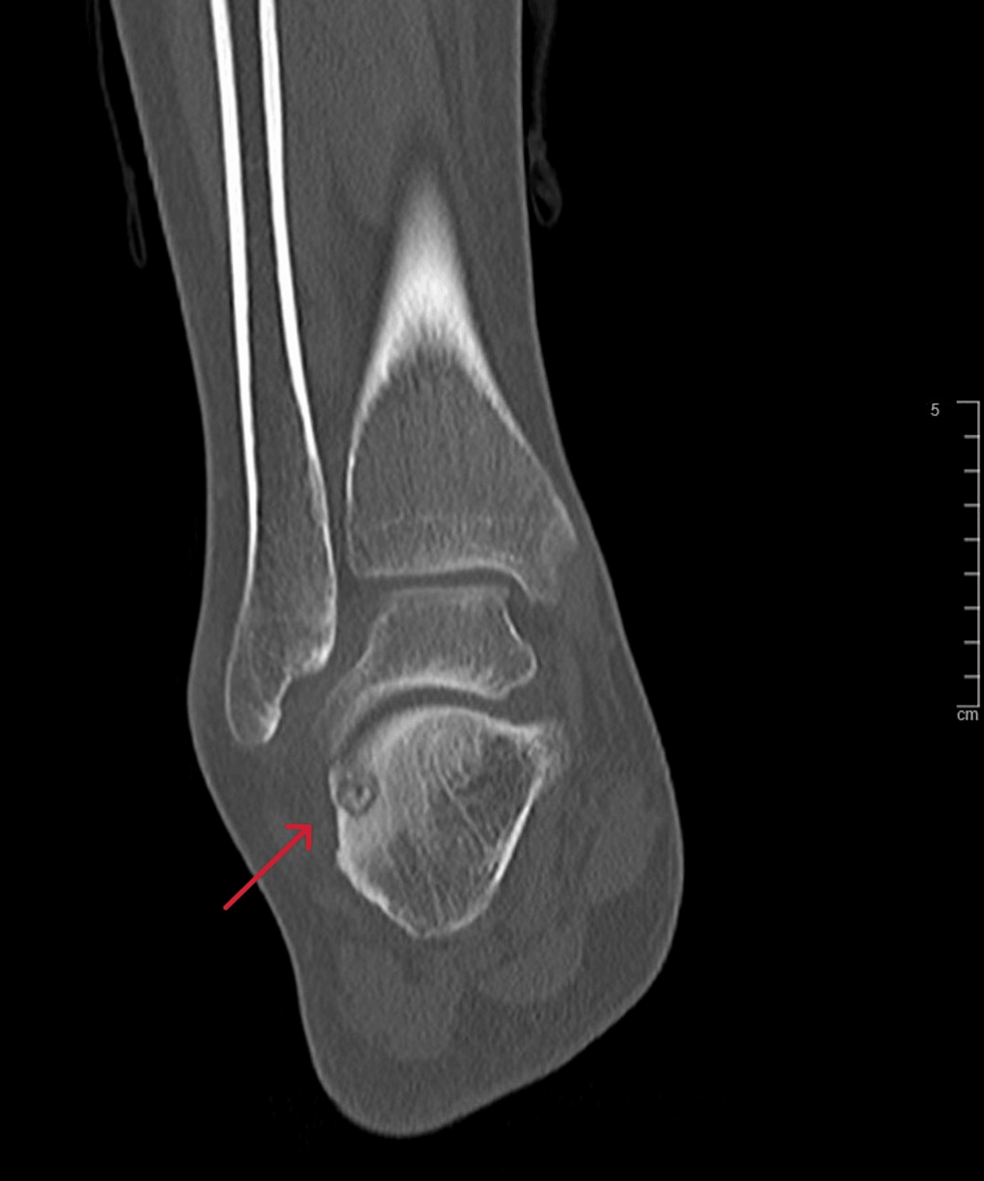
Coronal view CT scan of the right ankle at the time of presentation CT: computed tomography

**Figure 11:**
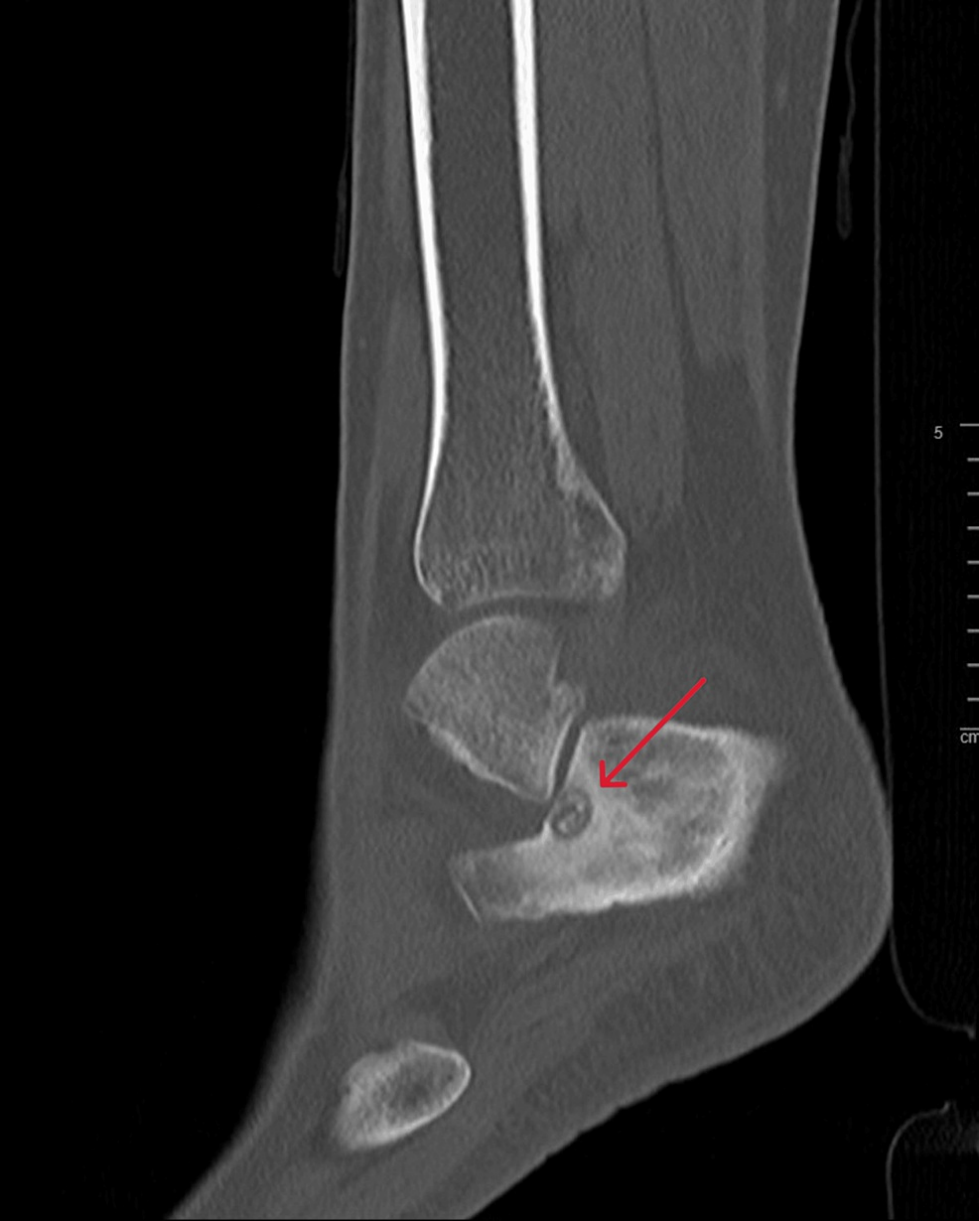
Sagittal view CT scan of the right ankle at the time of presentation CT: computed tomography

**Figure 12:**
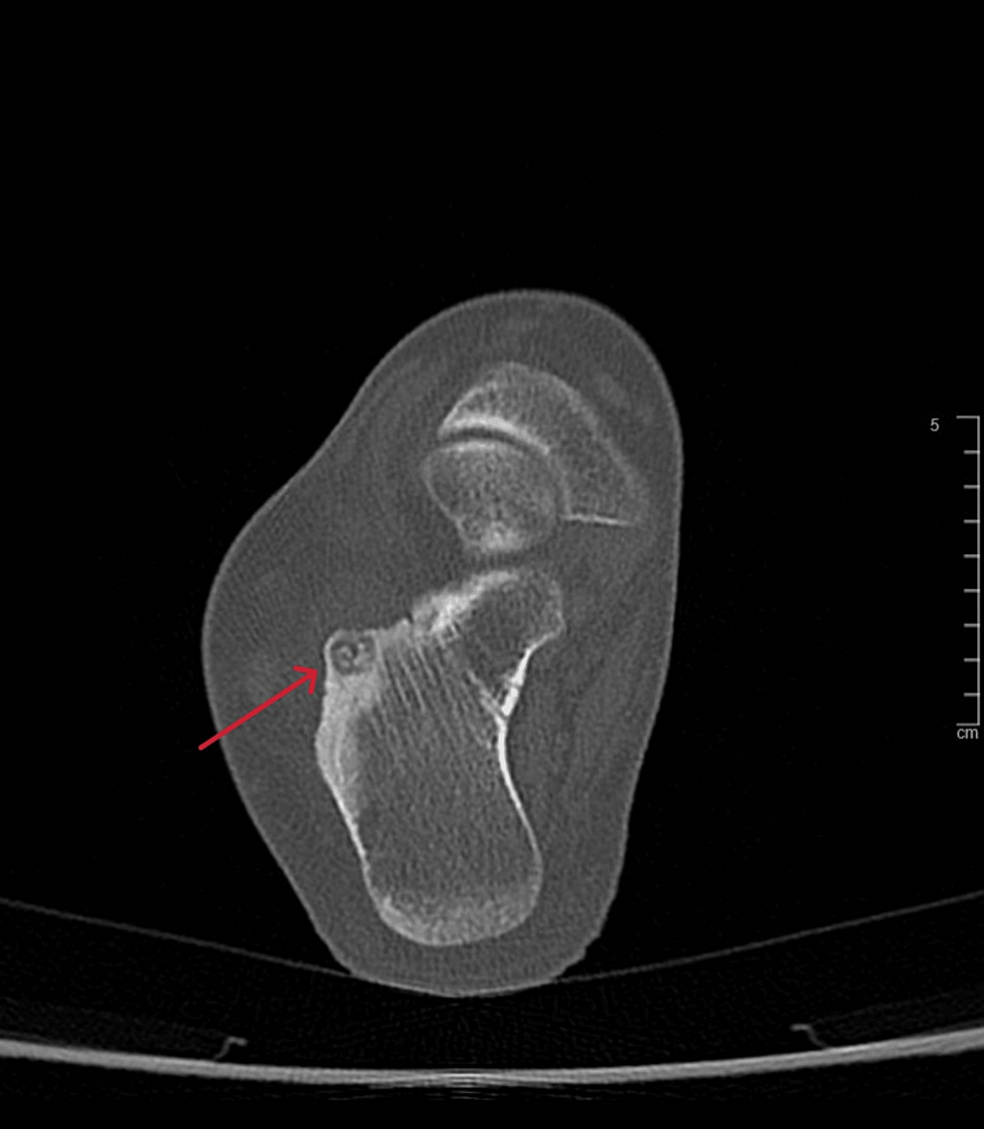
Axial view CT scan of the right ankle at the time of presentation CT: computed tomography

**Figure 13:**
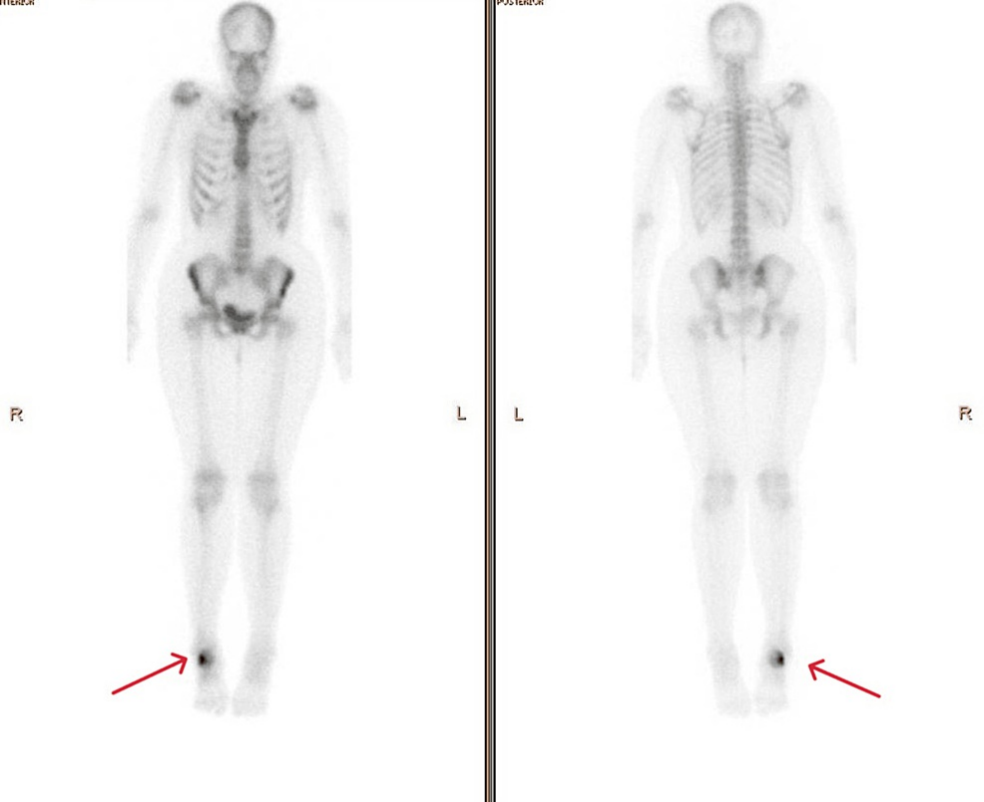
Whole-body bone scan at the time of presentation

A 21-year-old female college student presented with a three-year history of moderate pain in her heel with no previous history of trauma. The onset was insidious, and she had intermittent pain over the past three years. Her pain became more severe and persistent over months after a history of ankle sprain. The initial radiograph of her first presentation (Figures 1-3) after her ankle sprain did not show a fracture. A magnetic resonance imaging (MRI) for the ankle was requested (Figures 4-9) to assess her ankle ligaments and tendons, which showed incidental calcaneus changes that looked like nidus versus subchondral cyst. Computed tomography (CT) then was requested to confirm the diagnosis (Figures 10-12). Also, a bone scan was requested, which showed an intense hot area of focal uptake at the nidus and also showed low uptake in a reactive zone (double-density sign) (Figure 13).

On physical examination, the patient had a normal gait and mild swelling over the lateral aspect of her ankle with no overlying erythema. Bony tenderness was experienced by the patient upon palpation of the lateral aspect of the heel. She had a painless full range of motion of the ankle and subtalar joint and an intact distal neurovascular examination. The American Orthopedic Foot and Ankle Society (AOFAS) Ankle-Hindfoot Rating System was used to assess the clinical status of the ankle-hindfoot in this patient [6,7]. A score of 63 out of 100 was calculated at the time of presentation.

**Figure 14:**
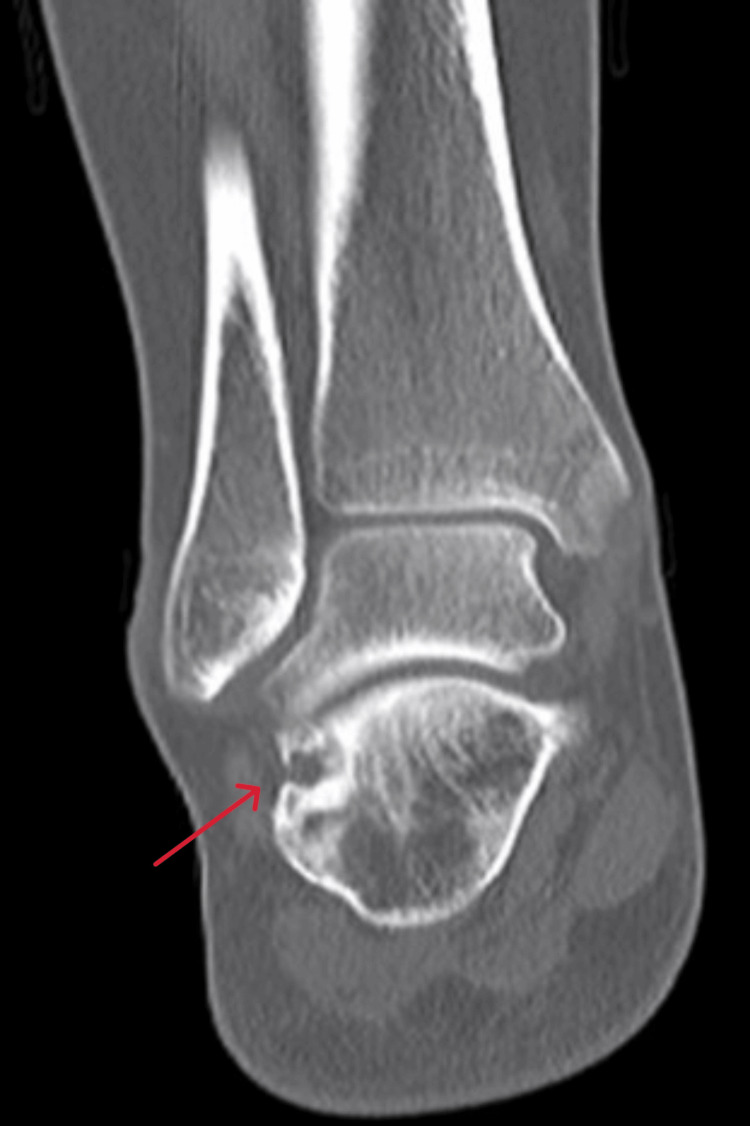
Coronal view CT scan of the right ankle at one-and-a-half-year follow-up CT: computed tomography

**Figure 15:**
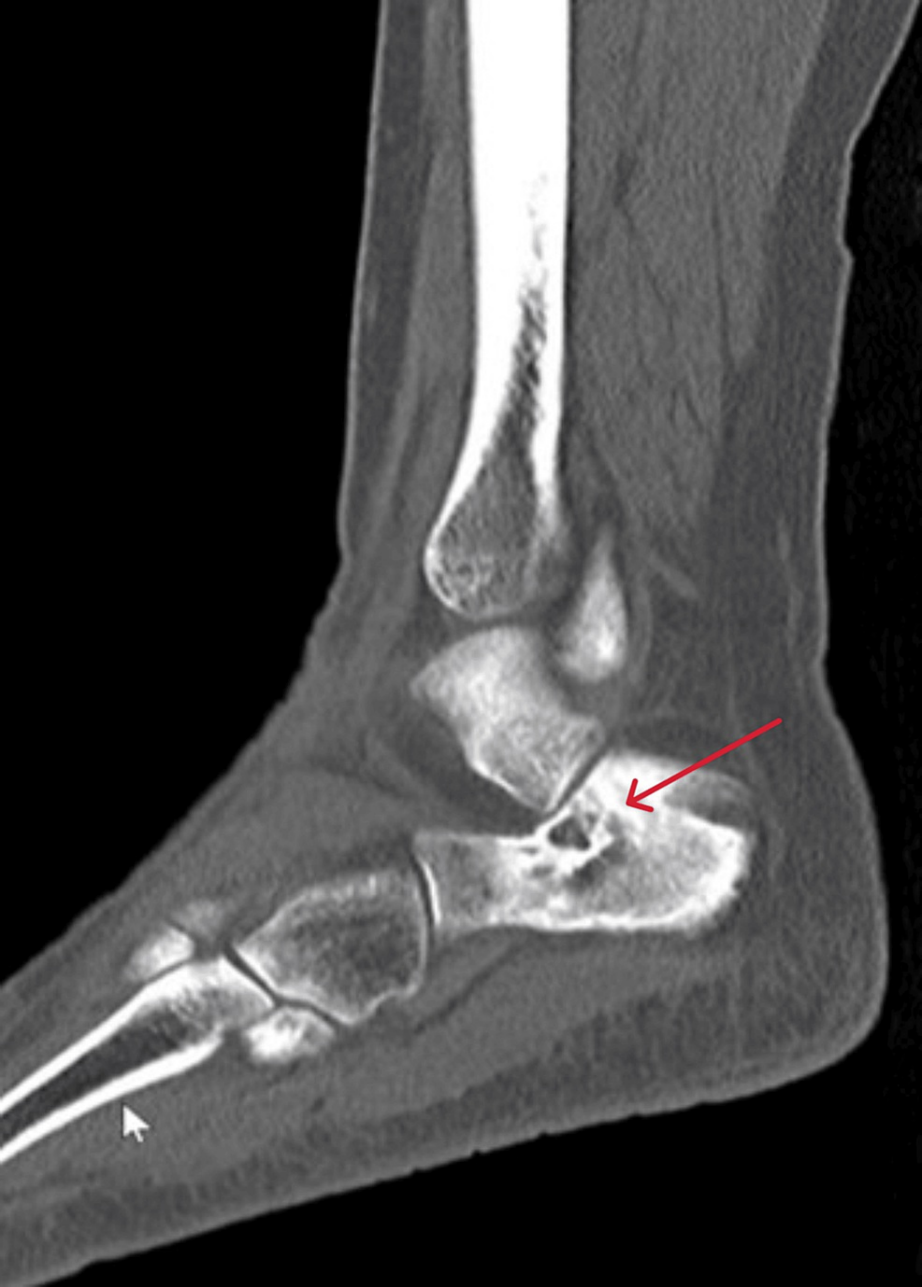
Sagittal view CT scan of the right ankle at one-and-a-half-year follow-up CT: computed tomography

**Figure 16:**
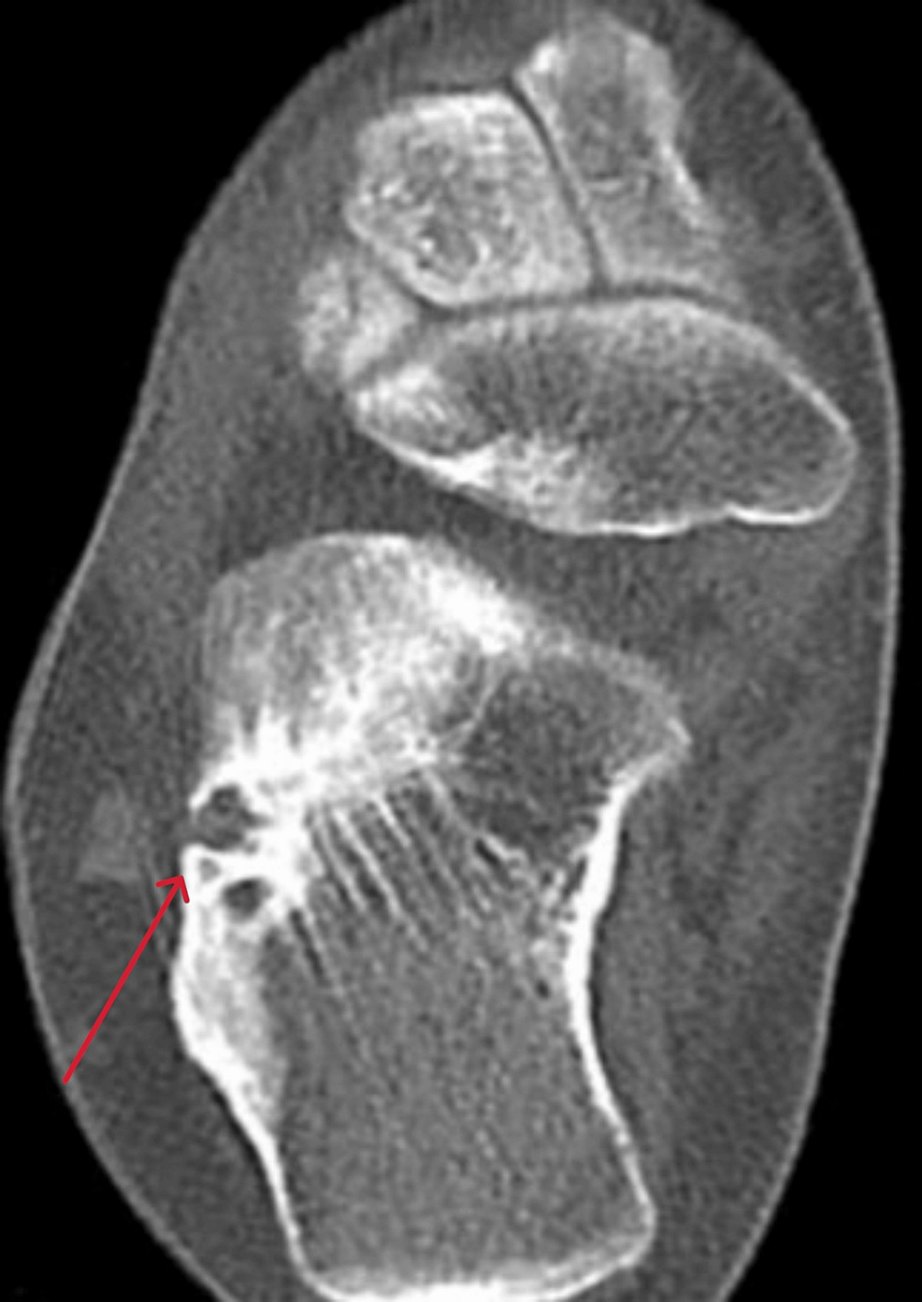
Axial view CT scan of the right ankle at one-and-a-half-year follow-up CT: computed tomography

Based on clinical examination and radiographic findings of osteoid osteoma, treatment options were fully explained to the patient, which included surgical resection and RFA. After further discussion, she opted to undergo RFA. Under general anesthesia, the patient was placed on the CT table. The lesion was imaged, and a bone biopsy needle was percutaneously introduced to obtain a tissue sample. A cortical bone and bone dust was reported by the pathologist, which was consistent with osteoid osteoma. The patient was kept non-weight-bearing immediately after the procedure for two weeks. At her one-month follow-up, she reported a complete resolution of pain. At a three-month follow-up, she had no evidence of recurrence and was pain-free. At one-and-a-half-year follow-up, the AOFAS Ankle-Hindfoot Rating was re-calculated, which was 100 out of 100, indicating good clinical status of the ankle and hindfoot postoperatively. Figures 14-16 show the CT scan images that were obtained one and a half years after the surgery.

## Discussion

Osteoid osteomas (OOs) are benign solitary lesions affecting predominantly long bones, causing cortical thickening. OOs comprise 10% of all benign bone tumors and 3% of all primary bone tumors affecting young adults and children with a male predominance with a 3:1 ratio [5,8,9].

Usually, osteoid osteomas are found in the femur, which is considered the most common location for it, followed by the spine, mainly in the posterior column [9], with only 4%-16% occurring in the foot, with the talus being the most affected at 31%-59% of cases. Calcaneal osteoid osteoma accounts for 12.5%-22% of cases. Regarding proximity from the joints, the specific location of osteoid osteomas is found mainly in two areas, either diaphyseal or metaphyseal, and, in some occasions, in the juxta-articular area and rarely in the epiphyseal. As for the medial to lateral location, it can be eccentric or concentric [10,11]. Multiple cases have reported osteoid osteomas located juxta- or intra-articular in the knee, elbow, hip, and ankle joints [12-18]. In contrast to the talus and calcaneum, lesions are usually subperiosteal or cancellous [5].

Osteoid osteoma patients have nocturnal pain in most cases, with unknown mechanism(s); pain is hypothesized to occur due to autonomic nerve fibers that run through the nidus, which are sensitive to vascular pressure changes [13]. It can be relieved by NSAIDs due to other mechanisms that are mainly blocking the cyclooxygenase (COX), which is responsible for increasing prostaglandins to 1,000-fold in the case of osteoid osteomas [14,15]. As for the worsening night pain, the exact process is yet to be explored. Regarding this case, it is not always worse at night, and osteoid osteoma pain could precede today’s pain. Other symptoms could be associated, including joint effusion, warmth, tenderness, stiffness, muscle atrophy, and joint degeneration, which many authors have stated could be the reason for misdiagnosis as inflammatory arthritis, chronic ankle sprain, osteomyelitis, and calcaneal stress fracture due to low suspicion of osteoid osteomas, hence delaying the exact diagnosis with a mean of two years and up to five years as in this case [16,17].

In this case, the patient’s symptoms have proceeded the finding of nidus and reactive sclerosis with other osteoid osteoma characteristics, and it was absent in X-ray, which usually happens and has been mentioned in the literature, especially if it is intra- or juxta-articular, with one case report mistakenly diagnosed mimicking os trigonum syndrome [18]. Such unusual locations for osteoid osteomas, such as the calcaneus, display less reactive sclerosis, which adds to the complexity of diagnosing these rare tumors.

On magnetic resonance imaging, the nidus may give a low or intermediate T1 signal and variable T2 signal and enhancement with contrast [19]. Yang et al. noticed that cases were misdiagnosed when an MRI was performed within three months of symptom onset [20]. This makes CT the best modality as it can detect small nidal calcification and early perinidal sclerosis. Osteoid osteoma can resolve spontaneously, as reported by Kneisl and Simon [21], and thus can be managed conservatively.

In general, treatment of osteoid osteomas ranges from conservative as self-limiting to surgical intervention, including RFA. Many studies have reported the need for treatment of osteoid osteomas in the calcaneum due to failed conservative management, whether it was RFA, arthroscopic surgery, or surgical excision for osteoid osteomas, and it has shown to yield good results [22-25].

The necessity of complete excision of osteoid osteomas to relieve pain has made open surgical resection a favorable treatment choice for many years [23]. However, in some studies, the small size of the nidus and difficulties in locating it led to incomplete excision of the nidus, with patients stating that their symptoms resolved [24].

RFA is an alternative method used to treat osteoid osteoma patients. Cantwell et al. conducted a study that included 200 patients with osteoid osteomas treated with RFA and showed success rates of 76%-100% [25]. Another study by Rosenthal et al. found no statistical difference in recurrence rates between those treated with surgical resection and those who had RFA, as both groups had an 8% incidence of recurrence. The study showed reduced cost, shorter hospital stays, and rehabilitation, allowing a quicker return to work and a lower risk of associated complications and morbidity for the RFA group [26]. Another study reported equivalent results with less pain, smaller incisions, and lower overall infection [27].

## Conclusions

In conclusion, calcaneal osteoid osteomas can be challenging to diagnose and treat as they might not present with classical nocturnal pain and radiographic reactive sclerosis. Thus, a high index of suspicion should be maintained for those who present with chronic pain and swelling and do not respond to conservative treatment. Percutaneous RFA is considered safe and effective for osteoid osteomas of the hindfoot and is associated with a shorter recovery period with similar results to surgical options.
